# Small RNA pathways in mammalian oocytes

**DOI:** 10.1002/2211-5463.70273

**Published:** 2026-05-19

**Authors:** Petr Svoboda, Josef Pasulka

**Affiliations:** ^1^ Institute of Molecular Genetics of the Czech Academy of Sciences Prague Czech Republic

**Keywords:** miRNA, oocyte, piRNA, retrotransposon, RNAi, siRNA

## Abstract

RNA silencing pathways use small RNAs to guide sequence‐specific repression of endogenous genes, mobile elements, and viruses. It has been 25 years since RNA interference (RNAi) was found in mouse oocytes and became the first identified small RNA pathway in mammals. Today, three distinct small RNA pathways are known to operate in mammalian oocytes—in addition to RNAi, there is the microRNA (miRNA) pathway and the PIWI‐associated RNA (piRNA) pathway. These pathways differ mechanistically and functionally and their co‐existence in the female germline evolved into different arrangements. This review aims to provide a basic introduction to mammalian RNA silencing pathways with a focus on mouse oocytes and key aspects of the pathways, which influence their biological roles in oocytes and zygotes, across mammals.

## Graphical abstract

Three distinct small RNA pathways operate in mammalian oocytes: RNAi interference (RNAi), the microRNA (miRNA) pathway, and the PIWI‐associated RNA (piRNA) pathway. These pathways use small RNAs to guide sequence‐specific repression and contribute to oocyte biology by targeting genes and mobile elements or appear insignificant since different arrangements evolved in different animals.

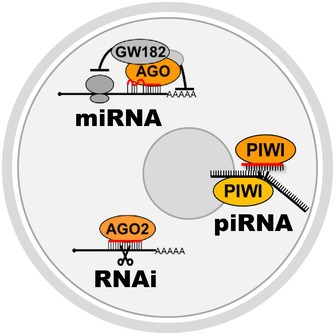

**Abbreviations**
AGO: Argonaute protein, AGO subfamily member
bp: base pair
DExD/H: Aspartate–Glutamate–undetermined–Aspartate/Histidine motif
DGCR8: DiGeorge syndrome critical region 8
dsRBD: double‐stranded RNA‐binding domain
dsRNA: double‐stranded RNA
endo‐siRNA: endogenous siRNA
ESC: embryonic stem cells
GW182: glycine‐tryptophan protein of 182 kDa
HEL1: helicase 1 subdomain of Dicer
lncRNA: long non‐coding RNA
miRISC: miRNA‐Induced Silencing Complex
miRNA: microRNA
mRNA: messenger RNA
MT: Mouse Transcript nonautonomous retrotranposon
MZT: maternal‐to‐zygotic transition
ncRNA: noncoding RNA
nt: nucleotide
PAZ domain: PIWI/Argonaute/Zwille domain
P‐body: processing body
piRNA: PIWI‐interacting RNA
PKR: protein kinase R
pre‐miRNA: precursor microRNA
pri‐miRNA: primary microRNA
RdRP: RNA‐dependent RNA polymerase
RISC: RNA‐induced silencing complex
RNA: ribonucleic acid
RNAi: RNA interference
RNase: ribonuclease
RNA seq: RNA sequencing
RPM: reads per million
siRNA: small interfering RNA
TARBP2: trans‐activation response element RNA‐binding protein 2
TE: transposable element

In RNA silencing pathways, small RNAs (20–30 nucleotides long) loaded on Argonaute family proteins guide sequence‐specific recognition of other RNAs and induce various transcriptional and post‐transcriptional effects. Although the first signs of eukaryotic small RNA pathways were observed in the early nineties [1, 2, 3], their research took off around the turn of the century.

The first discovered small RNA pathway in animals was the miRNA pathway [2, 3], which utilizes genome‐encoded miRNAs guiding post‐transcriptional gene repression. The miRNA pathway was discovered in *Caenorhabditis elegans* through a mutation causing a developmental timing defect. The mutation was mapped to a 22‐nt small RNA named *lin‐4* and its role was proposed to repress *lin‐14* mRNA through binding partially complementary sequences in its 3′ UTR [2, 3]. However, as *lin‐4* did not have homologs in more distantly related species, the omnipresence of animal miRNAs was not recognized until 2000 when was discovered *let‐7*, a miRNA expressed in somatic cells across Metazoa [4].

The second discovered animal small RNA pathway was RNAi, identified again in *C. elegans* as a sequence‐specific mRNA degradation response to long double‐stranded RNA (dsRNA) [5]. It was soon realized that RNAi is present in many animal groups including mammals, where it was first detected in mouse oocytes [6, 7, 8, 9, 10]. As we will discuss later, this was actually a serendipitous finding since mouse oocytes are the best adapted mammalian cell to execute RNAi.

The last discovered main RNA silencing pathway in animals was the piRNA pathway, which was recognized as a unique defense system of small RNAs mediating repression of transposable elements (TE) in the germline [11, 12, 13, 14, 15, 16]. The piRNA pathway is an equivalent of acquired immunity against active mobile elements and silences their targets at both post‐transcriptional and transcriptional levels (reviewed in [17]).

All three small RNA pathways (Fig. 1) were reported from mammalian oocytes but their co‐existence there is anything but stereotypical when inspecting an ever growing catalog of available small RNA‐sequencing (RNA‐seq) data from the literature [18, 19, 20, 21, 22, 23, 24, 25, 26, 27, 28, 29, 30]. Here, we will review the key features of the pathways and some of the interesting adaptations identified in mammalian oocytes.

**Fig. 1:**
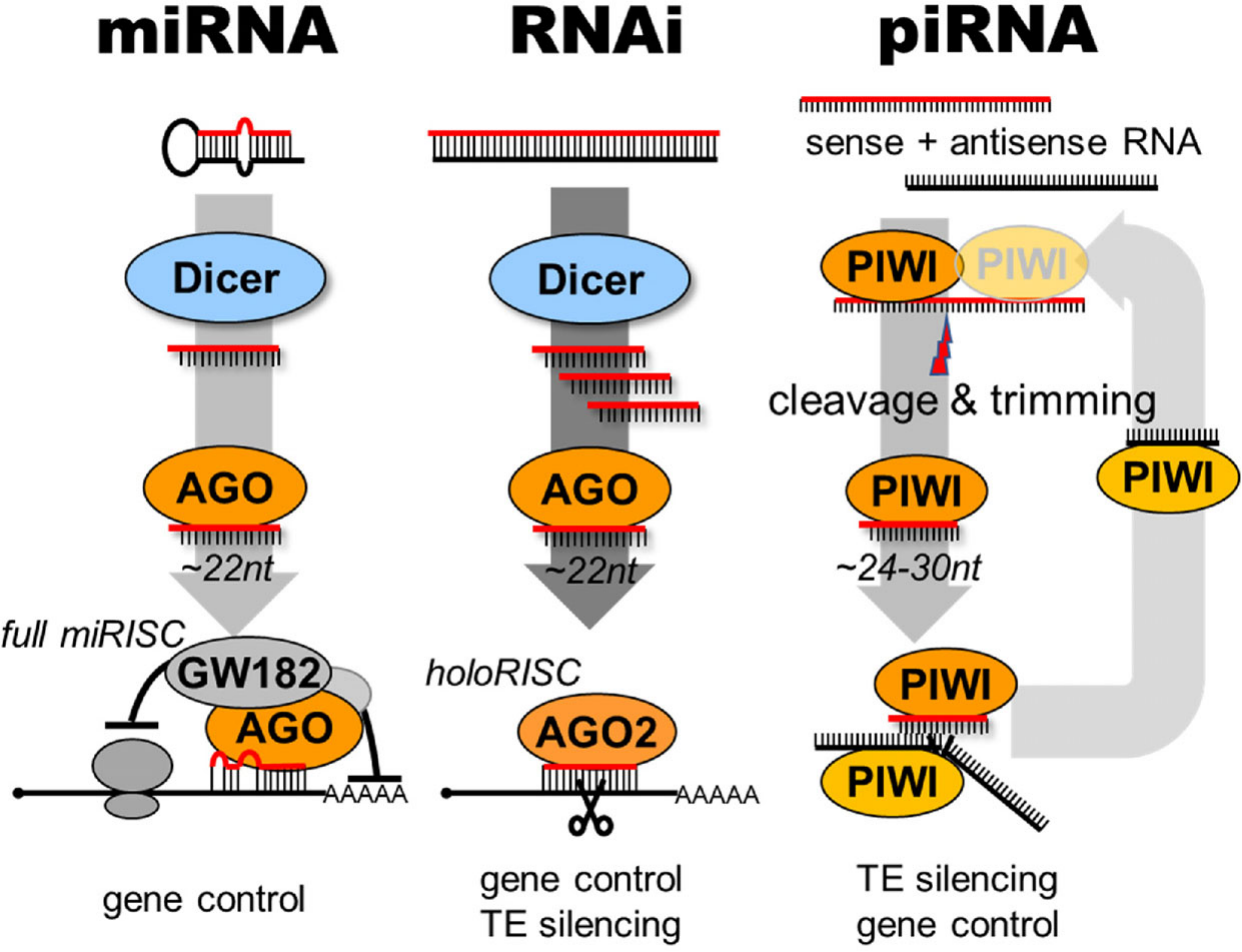
Schematic overview of the three main RNA silencing pathways in Metazoa.

## Maternal miRNAs and their (in)activity in mammalian oocytes

The miRNA pathway (reviewed in [31]) utilizes hundreds of genome‐encoded small RNAs, which have to be precisely excised from their small hairpin precursors in order to regulate gene expression. Long primary miRNA precursor transcripts (pri‐miRNA) are preprocessed in the nucleus into small hairpins (pre‐miRNA), which are transported to the cytoplasm and cleaved by RNase III Dicer to produce ~22‐nt‐long miRNAs loaded on the AGO subfamily of Argonaute proteins (AGO1‐4 in mammals). Animal miRNAs typically base‐pair imperfectly with 3′ UTRs of cognate mRNAs and induce translational repression and/or mRNA degradation. Notably, the silencing effect of a miRNA depends on the degree of base pairing and the AGO isoform. AGO2 is a functional endonuclease, which can execute cleavage of a cognate RNA when there is perfect complementarity with an AGO2‐bound small RNA [32, 33]. Other combinations of small RNAs and AGO paralogs result in translational repression followed by mRNA destabilization. This requires a fully formed effector complex (termed miRISC for miRNA‐Induced Silencing Complex) in which AGO2 interacts with an adaptor protein GW182, which recruits the rest of the complex [34, 35].

The miRNA pathway is the main mammalian small RNA pathway—miRNAs are the dominant small RNA class in somatic tissues and there is an ever‐growing list of their essential roles in different cells and biological processes (reviewed in [31]). Considering such widespread biological roles of miRNAs, it was initially assumed that miRNAs would also significantly regulate genes in mouse oocytes and this view influenced interpretation of oocyte‐specific Dicer knockouts phenotypes [36, 37]. However, it was noticed soon that mouse oocytes have relatively abundant endo‐siRNA [38], luciferase reporters for canonical miRNA‐mediated repression are not suppressed in fully grown mouse oocytes [39] and that mouse oocytes lacking an essential miRNA biogenesis factor DGCR8 develop normally and can even support development of viable offspring [40].

Importantly, the transcriptome of fully grown *Dgcr8* ^−/−^ oocytes is not significantly affected by the loss of miRNAs [40].

These observations raised questions: Why is miRNA‐mediated gene regulation not utilized by mouse oocytes? Is there any specific mechanism suppressing the miRNA pathway? Several lines of evidence showed that miRNAs function normally in mouse oocytes before the growth phase while the canonical miRNA‐mediated repression is lost on fully grown oocytes (∅ 80 μm). First, repression of partially complementary targets was observed in smaller meiotically incompetent oocytes (∅ 40 μm) before the onset of the growth phase [39]. Second, the onset of the growth phase was accompanied by the loss of processing bodies (P‐bodies), bright cytoplasmic foci where proteins of RNA metabolism, including miRISC, deadenylases, and decapping enzymes [41]. At the same time, miRNAs were detectable in fully grown oocytes by qPCR [36] and RNA sequencing [18, 38] and repression of perfectly complementary reporters in fully grown oocytes showed that miRNAs are still loaded onto AGO2.

We originally hypothesized that the miRNA pathway may be repressed at the level of formation of the full miRISC either by some inhibitory factor or by some of the components being a limiting factor. However, quantitative analysis of maternal miRNAs provided a simpler explanation. It was found that miRNAs do not accumulate like mRNAs during the oocyte growth because their turnover is not adapted to the diluting effect of the oocyte growth [42]. While maternal mRNAs have extended half‐lives, which facilitates their accumulation during growth phase (reviewed in [43]), half‐lives of maternal miRNAs are comparable to those in somatic cells [42]. Consequently, the most abundant miRNAs in growing and fully grown mouse oocytes are present in tens of thousands of molecules, which is a number similar to that observed in much smaller fibroblasts or HeLa cells [44]. This results in dilution of miRNAs to the picomolar range, in which miRNA can engage targets but are not abundant enough to mediate significant repression [42]. Notably, injection of 250 000 miRNA molecules was sufficient to restore reporter repression demonstrating that miRNA abundance is the key limiting factor. Since low miRNA concentrations were observed in rat, hamster, porcine, and bovine oocytes [19, 22, 42, 45, 46], it appears that miRNA inactivity is not just a mouse‐specific phenomenon but a common oocyte feature, which may even extend beyond mammals since lower abundance in oocytes appears consistent with zebrafish and Xenopus miRNA analyses [47, 48].

Importantly, there are exceptions to the rule. Detailed analysis of porcine and bovine oocytes showed that porcine *ssc‐miR‐205* and bovine *bta‐miR‐10b* resist the diluting effect of oocyte growth and retain the ability to efficiently suppress gene expression [45]. While the molecular mechanism underlying exceptional accumulation of these specific miRNAs (~1,6 million and ~ 261 000 molecules per oocyte of *ssc‐miR‐205* and bovine *bta‐miR‐10b*, respectively) remains unknown, functionality of these miRNAs at this abundance is consistent with the miRNA dilution hypothesis.

In any case, it is puzzling why oocytes would dilute miRNAs to insignificance. One possibility is that this is an early maternal‐to‐zygotic transition (MZT) reprogramming event. The oocyte miRNA profile looks rather somatic cell‐like (a third of maternal miRNAs in murine and other oocytes are *Let‐7* family miRNAs [18, 20, 22]). *Let‐7* miRNAs mark differentiated cells from *C. elegans* to humans and promote differentiation and loss of pluripotency in embryonic stem cells (ESCs, reviewed in [49]). *Let‐7* miRNAs would be rather unanticipated in mammalian oocytes considering the ability of oocyte cytoplasm to reprogram somatic nuclei into totipotency [50, 51]. Thus, miRNA dilution during MZT would eliminate negative effects of Let‐7 miRNAs and facilitate replacement of maternal miRNAs with zygotic miRNAs, particularly with the miR‐290 cluster, which is highly abundant in ESCs and counteracts *Let‐7* function [52].

Maternal miRNAs are globally degraded during MZT. Small RNA‐seq of full‐grown and meiotically mature porcine oocytes showed that demise of maternal miRNAs starts during meiotic maturation [53] while elimination of maternal miRNAs also appears to be induced by fertilization [36, Garcia‐Lopez, 2014 #413]. *Dgcr8* maternal and maternal/zygotic knockout shows that neither maternal nor zygotic miRNAs are required for ZGA, compaction, and blastocyst formation in mice [40]. Analysis of extremely abundant porcine miR‐205 suggested that it might be physiologically relevant for early development [45] but the analysis was done in parthenogenetic zygotes to avoid polyspermy. Analysis of the zebrafish model showed that miR‐430 miRNAs facilitate clearance of maternal mRNAs [54]. We would like to highlight two aspects of this result. First, the regulation is mediated by a uniquely adapted mechanism for producing a massive miRNA amount needed for miRNA‐mediated regulation–miR‐430 miRNAs in the genome form in a massive tandemly duplicated cluster, which becomes a transcriptional hotspot of the minor zygotic genome activation [55]. Notably, the miR‐430 is related to other vertebrate zygotic/pluripotent miRNAs sharing the same AAGUGC seed sequence [49]. The mammalian counterparts, pluripotent miRNAs of the miR‐290 and miR‐303 cluster [56] become expressed in mice during the zygotic genome activation (ZGA) and accumulate during early development [36] after the bulk of the maternal/zygotic mRNA exchange (reviewed in [43]). This implies that the role of zygotic miRNAs in maternal mRNA clearance is likely minimal.

## Unique gene and retrotransposon repression by maternal RNAi

RNAi has been originally defined as from long dsRNA‐induced sequence‐specific mRNA degradation [5], which is mediated by small RNAs generated from dsRNA by the RNase Dicer (Fig. 1). Mammalian RNAi is generally a residual ineffective pathway because it employs the same proteins, whose main role lies in the miRNA pathway. In particular, the mammalian Dicer efficiently processes miRNA precursors [57] but not long perfect duplexes because of the auto‐inhibitory effect of the N‐terminal helicase domain complex [58]. As a consequence, putative endo‐siRNAs are usually present in negligible amounts in RNA‐seq data from mammalian somatic cells. Furthermore, the innate immunity role of RNAi, which has been observed in invertebrates [59, 60], has been replaced by a sequence‐independent innate immunity response to dsRNA commonly referred to as the interferon response (reviewed in [61]). The interferon response effectively masks RNAi and some of the factors of the interferon response have inhibitory effects on RNAi [62, 63, 64, 65].

It was thus rather unexpected when microinjected long dsRNA induced a robust RNAi response in oocytes and zygotes [9, 10]. Apparently, mouse oocytes provide a suitable milieu for RNAi because they have suppressed the interferon response [66]. However, it also turned out that mouse oocytes are uniquely adapted and have highly active and essential endogenous RNAi. Small RNA‐seq showed that mouse oocytes contain endo‐siRNAs, which are more abundant than miRNAs [18, 38]. Mouse oocyte endo‐siRNAs include retrotransposon‐derived siRNAs as well as a unique class of gene‐regulating siRNAs derived from processed pseudogenes [18, 38]. The gene‐regulating siRNAs apparently originate from dsRNA, which forms when long noncoding RNAs (lncRNAs) containing antisense‐transcribed pseudogene sequences base pair with mRNAs [18, 38, 67]. It is likely that high stability of maternal poly(A) RNAs is an important factor facilitating such dsRNA formation.

In contrast to miRNAs and later‐described piRNAs, endo‐siRNAs are essential for proper meiotic maturation of mouse oocytes. As mentioned above, the loss of *Dgcr8* and canonical miRNAs has no obvious phenotype [40]. In contrast, the loss of *Dicer* and *Ago2* results in transcriptome changes and prominent spindle defects [36, 37, 68]. Furthermore, the catalytically inactive AGO2 mutant was produced to demonstrate the essential role of endonucleolytic cleavage of cognate maternal mRNAs [69]. Consistent with the spindle defect phenotype, putative endo‐siRNA targets are enriched in cell cycle regulators and genes involved in microtubule organization and dynamics [18].

**Fig. 2:**
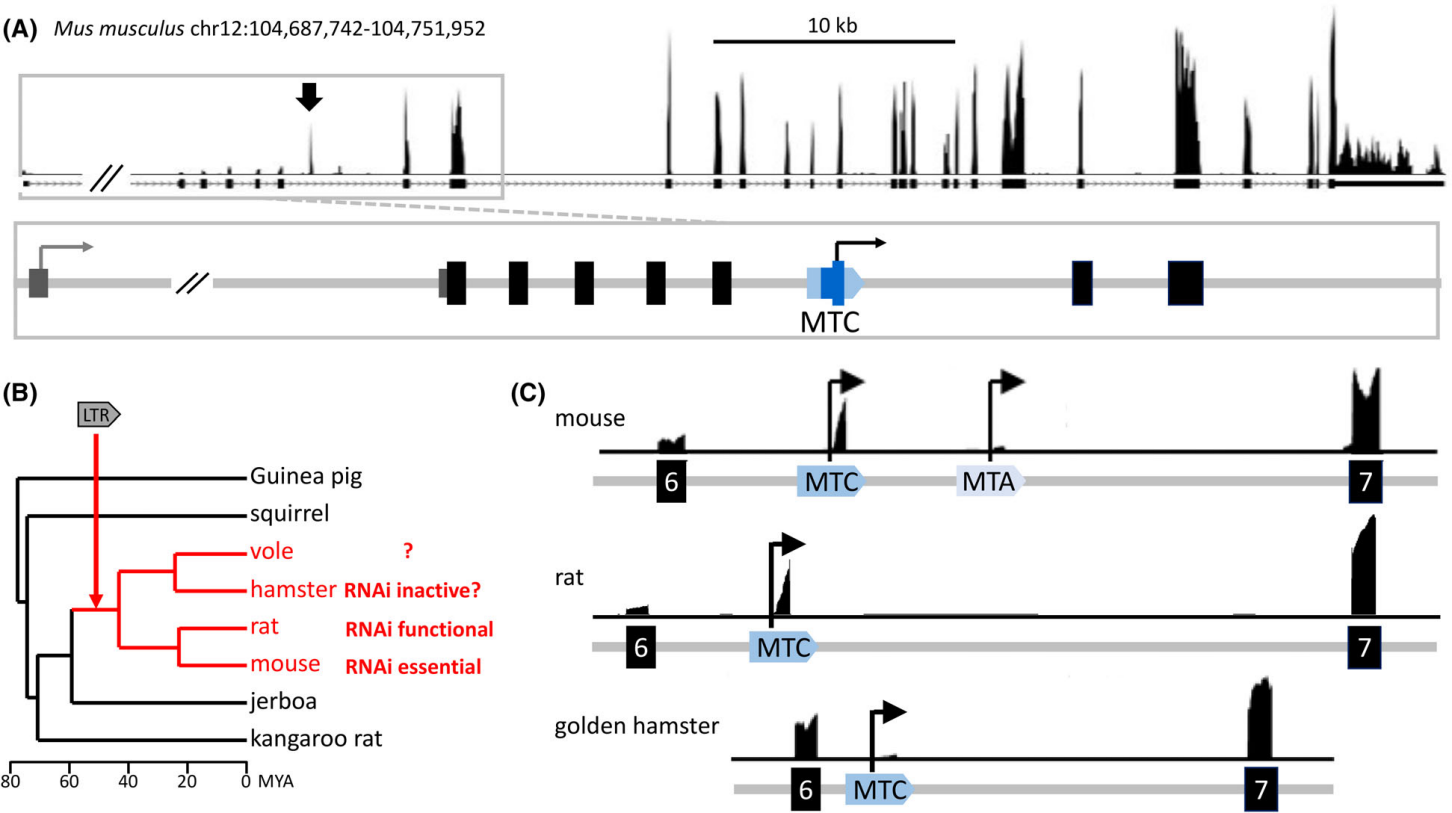
Dicer truncation supporting RNAi (A) An MT LTR element provides oocyte‐specific promoter for expression of Dicer^O^, a truncated Dicer isoform. At the top is shown a snapshot from the UCSC genome browser [93] with mapped oocyte RNA‐seq data [94], which reveals an alternative first exon (arrow). This exon originates from a transcription initiated from an intronic MT LTR insertion (MTC subfamily) depicted in blue in the scheme below. (B) Inspection of rodent genomes implies that the intronic MTC LTR insertion occurred in a common ancestor of mice and hamsters. The phylogenetic tree was constructed using the TimeTree of Life resource [95]. Whether RNAi operates in voles is unclear. RNAi is likely minimal in hamster oocytes as they have marginal expression of Dicer^O^ (see also panel C) and minimal levels of putative endo‐siRNAs [26, 29, 71]. Dicer^O^ expression in rat oocytes is stronger and endo‐siRNAs can be detected in small RNA‐seq data [29, 71]. (C) Different expression of Dicer^O^ in mouse, rat and golden hamster oocytes. Shown are snapshots from the UCSC genome browser [93] with mapped RNA‐seq data [71].

While several factors appear to contribute to the existence of robust endogenous RNAi in mouse oocytes, the key element is an adaptation of Dicer, which supports efficient siRNA production. Mouse oocytes express high levels of a unique Dicer isoform denoted Dicer^O^, which emerged in the rodents as a consequence of a mouse transcript (MT) LTR retrotransposon insertion into the Dicer gene (Fig. 2A) [70]. The MTC subfamily LTR insertion functions as an oocyte‐specific promoter, which drives expression of an N‐terminally truncated Dicer, which lacks the HEL1 helicase subdomain [70]. Consistent with earlier results [58], the truncated Dicer^O^ isoform shows higher activity *in vitro* and its ectopic expression in cells is sufficient to increase endo‐siRNA level. Importantly, deletion of the MTC LTR insertion from the genome results in the loss of Dicer^O^ and the same spindle defects observed in oocyte‐specific *Ago2* and *Dicer* knock‐outs [70].

The MTC LTR insertion supporting Dicer^O^ expression is present in genomes of the *Muridae* and *Cricetidae* rodent families (Fig. 2B), but hamster oocytes have low Dicer^O^ expression (Fig. 2C) [26, 29, 71]. Whether Dicer^O^ expression in hamsters was lost or enhanced during mouse and rat evolution is unclear, but generally low expression of young MT LTR insertions in mouse oocytes [71] implies that high expression of Dicer^O^ might be an additional adaptation. Notably, no similar truncated Dicer variant was found in transcriptomes of mammalian oocytes outside of *Muridae* and *Cricetidae* families (e.g [25, 26, 27, 46, 72, 73, 74].). There are also minimal levels of putative endo‐siRNA in non‐rodent oocytes [20, 21, 22, 23, 24, 25, 26, 27, 28, 29, 30]. However, it was shown that long dsRNA injection induced specific knock‐down effects in bovine [75], porcine [76], or ovine [77] oocytes. Apparently, when long dsRNA is abundant (10^5^–10^6^ molecules/oocyte), the less‐efficient cleavage by the full‐length Dicer is still sufficient to induce RNAi. This implies that mammalian oocytes lacking a strong interferon response have potential to evolve RNAi under favorable conditions, especially when adaptations supporting RNAi such as Dicer truncation would not be in conflict with the miRNA pathway (which is an issue in mouse somatic cells [78]). Therefore, it should not be ruled out that there may be another mammalian lineage where RNAi also became functionally important.

In any case, RNAi in mouse oocytes is an exceptional instance. First, antisense‐transcribed pseudogenes are transient sources of endo‐siRNAs during evolution since their base pairing with mRNAs *in trans* will be eroded by accumulating mutations. An example of such erosion was documented in *Sirena* lncRNA, which carries an *Elob* antisense pseudogene fragment. This pseudogene insertion occurred in the common ancestor of mice and rats. In rats, *Sirena* lncRNA lost the exon with the antisense sequence, while in mice, the full complementarity is largely disrupted, which negatively affects siRNA production along the pseudogene sequence [73]. Furthermore, even though rat oocytes also express Dicer^O^ (Fig. 2C), there are essentially no common gene‐regulating endo‐siRNAs between mouse and rat oocytes, as evidenced by comparison of small RNA sequencing data [29] or inspection of mouse‐rat synteny of antisense‐transcribed pseudogenes producing endo‐siRNAs in mouse oocytes–most of the antisense transcribed pseudogenes producing endo‐siRNAs in mouse oocytes are mouse‐specific [67, 79]. Consequently, deletion of the MT LTR driving Dicer^O^ expression in rat oocytes does not produce a meiotic spindle defect (Svoboda et al., unpublished results).

## piRNA, the workhorse of TE silencing of variable importance

The last small RNA silencing pathway reviewed here is the piRNA pathway. The piRNA pathway is distinct from the abovementioned pathways because piRNA biogenesis involves neither dsRNA nor Dicer and small RNAs are loaded onto the PIWI clade of the Argonaute protein family, which is primarily expressed during gametogenesis (reviewed in [80, 81]). piRNAs originate from discrete genomic regions (piRNA clusters) and come in two sorts: retrotransposon‐derived and those derived from other sequences. The first type functions to maintain genome integrity in the germline, whereas the role of the second type is being debated but may be important for selective gene control [82, 83].

**Fig. 3:**
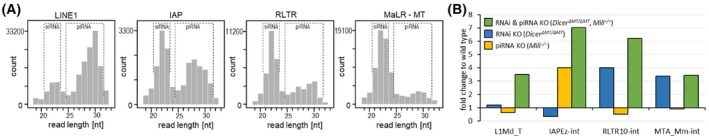
Functional overlap of RNAi and miRNA pathways in mouse oocytes. (A) Examples of small RNAs mapping to four selected classes or retrotransposons show variable ratios of putative siRNAs and piRNAs among the elements. Small RNA‐seq data were taken from [21]. (B) Functional analysis reveals selective and redundant transposable element repression by piRNA and RNAi pathways [79]. Shown are RNA level changes for selected well‐transcribed retrotransposon subfamilies in piRNA (*Mili* ^*−/−*^) and RNAi (*Dicer* ^*ΔMT/ΔMT*^) and double mutant.

In mice, three different PIWI proteins participate in piRNA biogenesis and function. All three are essential for spermatogenesis but nonessential for oogenesis [84, 85, 86]. Similarly, MOV10L1, a common piRNA biogenesis factor, is also essential for spermatogenesis but not oogenesis [87]. At the same time, analysis of primordial follicles and embryonic ovaries lacking piRNAs showed that maternal piRNAs suppress retrotransposons while this function is nonessential in oocytes [88, 89]. A possible explanation for the nonessential function of piRNAs during mouse oocyte development is that loss of piRNAs is compensated by a highly active RNAi pathway. This notion is supported by the small RNA profile from mouse oocytes [21] suggesting that LINE1 and IAP, two autonomous TEs mobilized in male sterile mutants [90, 91] may be targeted by both piRNAs and siRNAs (Fig. 3A). Redundant targeting of LINE1 and IAP was observed in mutants lacking piRNA, RNAi, or both pathways (Fig. 3B) where maximum derepression of full‐length intact LINE and IAP elements was observed in double mutants [79]. At the same time, there was no evidence for mobilization of these TEs [79]. It thus remains unknown whether the piRNA pathway in the murine female germline is dispensable because it is compensated by highly active RNAi. Unfortunately, the meiotic spindle defect caused by the loss of the RNAi pathway prevents investigating whether RNAi masks a postzygotic phenotype caused by the absence of the piRNA pathway.

For a while, mouse mutant data were defining the mammalian female germline piRNA narrative. However, small RNA sequencing from other mammalian oocytes suggested that the piRNA pathway in mammalian oocytes may be more active and functionally significant than the mouse data suggested [20, 23]. For example, in human, oocytes was found a unique class of ~20‐nt piRNAs associated with PIWIL3 protein expressed primarily in the female germline [23]. *Piwil3* gene was lost during mouse evolution, but it is present in other mammals including hamsters. In 2021, three publications reported phenotypes of knockout of piRNA pathway components *MOV10L1*, *PIWIL1*, and *PIWIL3* in golden hamsters and revealed that the piRNA pathway is essential for fertility on both hamster sexes [25, 26, 27]. The loss of *MOV10L1* and *PIWIL1* in the female germline caused postmeiotic and postzygotic sterility while the loss of *PIWIL3* caused subfertility [25, 26, 27]. In the male germline, the onset of the *MOV10L1* null phenotype occurred earlier than in mice and was accompanied with upregulated expression of MYSERV, an LTR element expressed in hamsters before establishment of spermatogonia [26]. Further analysis showed nonredundant functions of PIWIL1 and PIWIL3 in golden hamster gametogenesis [92]. Remarkably, knockout of *PIWIL3* in rabbits showed that PIWIL3 is essential for female fertility as embryos lacking maternal PIWIL3 arrest developmentally at the 8‐cell stage [30]. Altogether, these data demonstrate that functional significance of the piRNA pathway in mammalian oocytes varies between species and that mice are rather a derived model.

Finally, there is one important aspect of the piRNA pathway, which must be highlighted. The piRNA pathway is an adaptive mechanism primarily supporting TE silencing in the germline, which may also adopt gene‐regulating function. Mutant phenotypes uncover such functionally important adaptations concerning recognition and silencing of specific TEs during the germline cycle or stochastically acquired important gene regulation. This readily explains why piRNA pathway defects would cause such diversity of phenotypes in different mammals. Or no phenotypes such as in the female germline in mice. Notably, analysis of piRNAs in the 13‐lined ground squirrel implies that the piRNA pathway is relaxed there due to the lack of mobilizing transposable elements [28].

## Conclusions

**Fig. 4:**
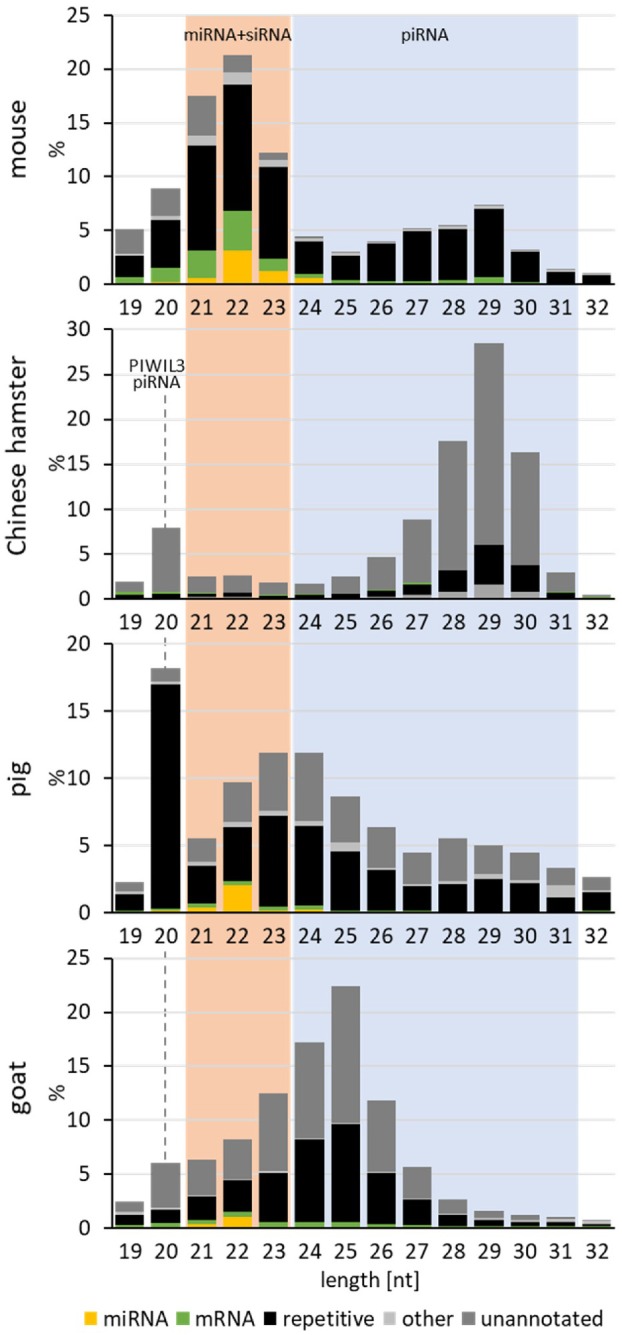
Examples of small RNAs from oocytes of four mammalian species from published data [29] illustrate the diversity of miRNA, siRNA and piRNA presence in oocytes. The 20 nt peak in Chinese hamster, pig and goat oocytes would correspond to 20 nt PIWIL3‐associated piRNAs [23] (mice lack PIWIL3). Mouse oocytes are known to contain abundant 21–23 nt endo‐siRNAs originating from complementary mRNAs, long ncRNA and transcripts from transposable elements produced by a specialized variant of Dicer. Other oocytes are not likely to contain relevant amounts of endo‐siRNAs; 21–23 nt reads may be of piRNA origin. Given the variable quality of genome annotation, unannotated sequences may still contain repetitive elements. Fractions labeled as “other” belong to other annotated RNAs (rRNA, tRNA and other ncRNAs).

Over two decades of functional genomics analyses of RNA silencing in the mouse model have revealed that while the mouse is an outstanding model to study RNA silencing in oocytes, it also poorly represents mammalian oocytes when it comes to RNA silencing. The oddity of the mouse is apparently just one example in the existing diversity of small RNA populations harbored by different mammalian oocytes (Fig. 4). These examples from a comprehensive study of small RNAs from oocytes of 11 different mammals illustrate this remarkable diversity in terms of abundance and composition of different classes of small RNAs [29]. It holds a promise that there are many interesting functional adaptations of small RNA pathways in mammalian oocytes yet to be uncovered.

## Conflicts of interest

The authors declare no conflicts of interest.

## Author contributions

PS wrote the manuscript and designed figures. JP did bioinformatics analyses and revised the manuscript.

## Data Availability

All data presented in the article were obtained from public resources and their original published sources are referenced in the text.

## References

[1] Napoli C , Lemieux C and Jorgensen R (1990) Introduction of a chimeric Chalcone synthase gene into petunia results in reversible Co‐suppression of homologous genes in trans. Plant Cell 2, 279–289. 12354959 10.1105/tpc.2.4.279 PMC159885

[2] Lee RC , Feinbaum RL and Ambros V (1993) The *C. elegans* heterochronic gene lin‐4 encodes small RNAs with antisense complementarity to lin‐14. Cell 75, 843–854. 8252621 10.1016/0092-8674(93)90529-y

[3] Wightman B , Ha I and Ruvkun G (1993) Posttranscriptional regulation of the heterochronic gene lin‐14 by lin‐4 mediates temporal pattern formation in C. Elegans. Cell 75, 855–862. 8252622 10.1016/0092-8674(93)90530-4

[4] Reinhart BJ , Slack FJ , Basson M , Pasquinelli AE , Bettinger JC , Rougvie AE , Horvitz HR and Ruvkun G (2000) The 21‐nucleotide let‐7 RNA regulates developmental timing in Caenorhabditis elegans. Nature 403, 901–906. 10706289 10.1038/35002607

[5] Fire A , Xu S , Montgomery MK , Kostas SA , Driver SE and Mello CC (1998) Potent and specific genetic interference by double‐stranded RNA in Caenorhabditis elegans. Nature 391, 806–811. 9486653 10.1038/35888

[6] Sanchez Alvarado A and Newmark PA (1999) Double‐stranded RNA specifically disrupts gene expression during planarian regeneration. Proc Natl Acad Sci USA 96, 5049–5054. 10220416 10.1073/pnas.96.9.5049 PMC21814

[7] Misquitta L and Paterson BM (1999) Targeted disruption of gene function in drosophila by RNA interference (RNA‐i): a role for nautilus in embryonic somatic muscle formation. Proc Natl Acad Sci USA 96, 1451–1456. 9990044 10.1073/pnas.96.4.1451 PMC15483

[8] Lohmann JU , Endl I and Bosch TC (1999) Silencing of developmental genes in hydra. Dev Biol 214, 211–214. 10491269 10.1006/dbio.1999.9407

[9] Wianny F and Zernicka‐Goetz M (2000) Specific interference with gene function by double‐stranded RNA in early mouse development. Nat Cell Biol 2, 70–75. 10655585 10.1038/35000016

[10] Svoboda P , Stein P , Hayashi H and Schultz RM (2000) Selective reduction of dormant maternal mRNAs in mouse oocytes by RNA interference. Development 127, 4147–4156. 10976047 10.1242/dev.127.19.4147

[11] Aravin A , Gaidatzis D , Pfeffer S , Lagos‐Quintana M , Landgraf P , Iovino N , Morris P , Brownstein MJ , Kuramochi‐Miyagawa S , Nakano T *et al*. (2006) A novel class of small RNAs bind to MILI protein in mouse testes. Nature 442, 203–207. 16751777 10.1038/nature04916

[12] Girard A , Sachidanandam R , Hannon GJ and Carmell MA (2006) A germline‐specific class of small RNAs binds mammalian Piwi proteins. Nature 442, 199–202. 16751776 10.1038/nature04917

[13] Grivna ST , Beyret E , Wang Z and Lin H (2006) A novel class of small RNAs in mouse spermatogenic cells. Genes Dev 20, 1709–1714. 16766680 10.1101/gad.1434406 PMC1522066

[14] Aravin AA , Hannon GJ and Brennecke J (2007) The Piwi‐piRNA pathway provides an adaptive defense in the transposon arms race. Science 318, 761–764. 17975059 10.1126/science.1146484

[15] Aravin AA , Sachidanandam R , Girard A , Fejes‐Toth K and Hannon GJ (2007) Developmentally regulated piRNA clusters implicate MILI in transposon control. Science 316, 744–747. 17446352 10.1126/science.1142612

[16] Brennecke J , Aravin AA , Stark A , Dus M , Kellis M , Sachidanandam R and Hannon GJ (2007) Discrete small RNA‐generating loci as master regulators of transposon activity in drosophila. Cell 128, 1089–1103. 17346786 10.1016/j.cell.2007.01.043

[17] Czech B , Munafo M , Ciabrelli F , Eastwood EL , Fabry MH , Kneuss E and Hannon GJ (2018) piRNA‐guided genome defense: from biogenesis to silencing. Annu Rev Genet 52, 131–157. 30476449 10.1146/annurev-genet-120417-031441 PMC10784713

[18] Tam OH , Aravin AA , Stein P , Girard A , Murchison EP , Cheloufi S , Hodges E , Anger M , Sachidanandam R , Schultz RM *et al*. (2008) Pseudogene‐derived small interfering RNAs regulate gene expression in mouse oocytes. Nature 453, 534–538. 18404147 10.1038/nature06904 PMC2981145

[19] Garcia‐Lopez J , Hourcade Jde D , Alonso L , Cardenas DB and del Mazo J (2014) Global characterization and target identification of piRNAs and endo‐siRNAs in mouse gametes and zygotes. Biochim Biophys Acta 1839, 463–475. 24769224 10.1016/j.bbagrm.2014.04.006

[20] Roovers EF , Rosenkranz D , Mahdipour M , Han CT , He N , de Sousa C , Lopes SM , van der Westerlaken LA , Zischler H , Butter F *et al*. (2015) Piwi proteins and piRNAs in mammalian oocytes and early embryos. Cell Rep 10, 2069–2082. 25818294 10.1016/j.celrep.2015.02.062

[21] Yang Q , Lin J , Liu M , Li R , Tian B , Zhang X , Xu B , Liu M , Zhang X , Li Y *et al*. (2016) Highly sensitive sequencing reveals dynamic modifications and activities of small RNAs in mouse oocytes and early embryos. Sci Adv 2, e1501482. 27500274 10.1126/sciadv.1501482 PMC4974095

[22] Gad A , Nemcova L , Murin M , Kanka J , Laurincik J , Benc M , Pendovski L and Prochazka R (2019) microRNA expression profile in porcine oocytes with different developmental competence derived from large or small follicles. Mol Reprod Dev 86, 426–439. 30756429 10.1002/mrd.23121

[23] Yang Q , Li R , Lyu Q , Hou L , Liu Z , Sun Q , Liu M , Shi H , Xu B , Yin M *et al*. (2019) Single‐cell CAS‐seq reveals a class of short PIWI‐interacting RNAs in human oocytes. Nat Commun 10, 3389. 31358756 10.1038/s41467-019-11312-8 PMC6662892

[24] Paloviita P , Hyden‐Granskog C , Yohannes DA , Paluoja P , Kere J , Tapanainen JS , Krjutskov K , Tuuri T , Vosa U and Vuoristo S (2021) Small RNA expression and miRNA modification dynamics in human oocytes and early embryos. Genome Res 31, 1474–1485. 34340992 10.1101/gr.268193.120 PMC8327922

[25] Hasuwa H , Iwasaki YW , Au Yeung WK , Ishino K , Masuda H , Sasaki H and Siomi H (2021) Production of functional oocytes requires maternally expressed PIWI genes and piRNAs in golden hamsters. Nat Cell Biol 23, 1002–1012. 34489571 10.1038/s41556-021-00745-3

[26] Loubalova Z , Fulka H , Horvat F , Pasulka J , Malik R , Hirose M , Ogura A and Svoboda P (2021) Formation of spermatogonia and fertile oocytes in golden hamsters requires piRNAs. Nat Cell Biol 23, 992–1001. 34489573 10.1038/s41556-021-00746-2 PMC8437802

[27] Zhang H , Zhang F , Chen Q , Li M , Lv X , Xiao Y , Zhang Z , Hou L , Lai Y , Zhang Y *et al*. (2021) The piRNA pathway is essential for generating functional oocytes in golden hamsters. Nat Cell Biol 23, 1013–1022. 34489574 10.1038/s41556-021-00750-6

[28] Vandewege MW , Patt RN 2nd , Merriman DK , Ray DA and Hoffmann FG (2022) The PIWI/piRNA response is relaxed in a rodent that lacks mobilizing transposable elements. RNA 28, 609–621. 35064043 10.1261/rna.078862.121 PMC8925971

[29] Hou L , Liu W , Zhang H , Li R , Liu M , Shi H and Wu L (2024) Divergent composition and transposon‐silencing activity of small RNAs in mammalian oocytes. Genome Biol 25, 80. 38532500 10.1186/s13059-024-03214-w PMC10964541

[30] Gong Y , Li L , Qian Y , Lu T , Zhang Z , Jiang L , Liu G , Cui M , Li S , Li Z *et al*. (2025) “PIWIL3‐piRNA pathway is essential for rabbit oogenesis and embryogenesis via broad regulation of the transcriptome and proteome, *bioRxiv*, 2025.10.23.684072.” 10.1038/s41467-026-73503-4 PMC13389465 42192102

[31] Bartel DP (2018) Metazoan MicroRNAs. Cell 173, 20–51. 29570994 10.1016/j.cell.2018.03.006 PMC6091663

[32] Hutvagner G and Zamore PD (2002) A microRNA in a multiple‐turnover RNAi enzyme complex. Science 297, 2056–2060. 12154197 10.1126/science.1073827

[33] Meister G , Landthaler M , Patkaniowska A , Dorsett Y , Teng G and Tuschl T (2004) Human Argonaute2 mediates RNA cleavage targeted by miRNAs and siRNAs. Mol Cell 15, 185–197. 15260970 10.1016/j.molcel.2004.07.007

[34] Liu J , Rivas FV , Wohlschlegel J , Yates JR 3rd , Parker R and Hannon GJ (2005) A role for the P‐body component GW182 in microRNA function. Nat Cell Biol 7, 1261–1266. 16284623 10.1038/ncb1333 PMC1804202

[35] Rehwinkel J , Behm‐Ansmant I , Gatfield D and Izaurralde E (2005) A crucial role for GW182 and the DCP1:DCP2 decapping complex in miRNA‐mediated gene silencing. RNA 11, 1640–1647. 16177138 10.1261/rna.2191905 PMC1370850

[36] Tang F , Kaneda M , O'Carroll D , Hajkova P , Barton SC , Sun YA , Lee C , Tarakhovsky A , Lao K and Surani MA (2007) Maternal microRNAs are essential for mouse zygotic development. Genes Dev 21, 644–648. 17369397 10.1101/gad.418707 PMC1820938

[37] Murchison EP , Stein P , Xuan Z , Pan H , Zhang MQ , Schultz RM and Hannon GJ (2007) Critical roles for dicer in the female germline. Genes Dev 21, 682–693. 17369401 10.1101/gad.1521307 PMC1820942

[38] Watanabe T , Totoki Y , Toyoda A , Kaneda M , Kuramochi‐Miyagawa S , Obata Y , Chiba H , Kohara Y , Kono T , Nakano T *et al*. (2008) Endogenous siRNAs from naturally formed dsRNAs regulate transcripts in mouse oocytes. Nature 453, 539–543. 18404146 10.1038/nature06908

[39] Ma J , Flemr M , Stein P , Berninger P , Malik R , Zavolan M , Svoboda P and Schultz RM (2010) MicroRNA activity is suppressed in mouse oocytes. Curr Biol 20, 265–270. 20116252 10.1016/j.cub.2009.12.042 PMC2824427

[40] Suh N , Baehner L , Moltzahn F , Melton C , Shenoy A , Chen J and Blelloch R (2010) MicroRNA function is globally suppressed in mouse oocytes and early embryos. Curr Biol 20, 271–277. 20116247 10.1016/j.cub.2009.12.044 PMC2872512

[41] Flemr M , Ma J , Schultz RM and Svoboda P (2010) P‐body loss is concomitant with formation of a messenger RNA storage domain in mouse oocytes. Biol Reprod 82, 1008–1017. 20075394 10.1095/biolreprod.109.082057 PMC2857638

[42] Kataruka S , Modrak M , Kinterova V , Malik R , Zeitler DM , Horvat F , Kanka J , Meister G and Svoboda P (2020) MicroRNA dilution during oocyte growth disables the microRNA pathway in mammalian oocytes. Nucleic Acids Res 48, 8050–8062. 32609824 10.1093/nar/gkaa543 PMC7430632

[43] Svoboda P , Franke V and Schultz RM (2015) In Sculpting the Transcriptome During the Oocyte‐to‐Embryo Transition in Mouse in Maternal‐to‐Zygotic Transition ( Lipshitz HD , ed.), pp. 305–349. Academic Press, London, UK. 10.1016/bs.ctdb.2015.06.004 26358877

[44] Bosson AD , Zamudio JR and Sharp PA (2014) Endogenous miRNA and target concentrations determine susceptibility to potential ceRNA competition. Mol Cell 56, 347–359. 25449132 10.1016/j.molcel.2014.09.018 PMC5048918

[45] Kataruka S , Kinterova V , Horvat F , Kulmann MIR , Kanka J and Svoboda P (2022) Physiologically relevant miRNAs in mammalian oocytes are rare and highly abundant. EMBO Rep 23, e53514. 34866300 10.15252/embr.202153514 PMC8811628

[46] Graf A , Krebs S , Zakhartchenko V , Schwalb B , Blum H and Wolf E (2014) Fine mapping of genome activation in bovine embryos by RNA sequencing. Proc Natl Acad Sci USA 111, 4139–4144. 24591639 10.1073/pnas.1321569111 PMC3964062

[47] Chen PY , Manninga H , Slanchev K , Chien M , Russo JJ , Ju J , Sheridan R , John B , Marks DS , Gaidatzis D *et al*. (2005) The developmental miRNA profiles of zebrafish as determined by small RNA cloning. Genes Dev 19, 1288–1293. 15937218 10.1101/gad.1310605 PMC1142552

[48] Tang GQ and Maxwell ES (2008) Xenopus microRNA genes are predominantly located within introns and are differentially expressed in adult frog tissues via post‐transcriptional regulation. Genome Res 18, 104–112. 18032731 10.1101/gr.6539108 PMC2134782

[49] Svoboda P and Flemr M (2010) The role of miRNAs and endogenous siRNAs in maternal‐to‐zygotic reprogramming and the establishment of pluripotency. EMBO Rep 11, 590–597. 20651740 10.1038/embor.2010.102 PMC2920432

[50] Campbell KH , McWhir J , Ritchie WA and Wilmut I (1996) Sheep cloned by nuclear transfer from a cultured cell line. Nature 380, 64–66. 8598906 10.1038/380064a0

[51] Gao S , McGarry M , Latham KE and Wilmut I (2003) Cloning of mice by nuclear transfer. Cloning Stem Cells 5, 287–294. 14733747 10.1089/153623003772032790

[52] Melton C , Judson RL and Blelloch R (2010) Opposing microRNA families regulate self‐renewal in mouse embryonic stem cells. Nature 463, 621–626. 20054295 10.1038/nature08725 PMC2894702

[53] Yang CX , Du ZQ , Wright EC , Rothschild MF , Prather RS and Ross JW (2012) Small RNA profile of the cumulus‐oocyte complex and early embryos in the pig. Biol Reprod 87, 117. 22933518 10.1095/biolreprod.111.096669

[54] Giraldez AJ , Mishima Y , Rihel J , Grocock RJ , Van Dongen S , Inoue K , Enright AJ and Schier AF (2006) Zebrafish MiR‐430 promotes deadenylation and clearance of maternal mRNAs. Science 312, 75–79. 16484454 10.1126/science.1122689

[55] Hadzhiev Y , Wheatley L , Cooper L , Ansaloni F , Whalley C , Chen Z , Finaurini S , Gustincich S , Sanges R , Burgess S *et al*. (2023) The miR‐430 locus with extreme promoter density forms a transcription body during the minor wave of zygotic genome activation. Dev Cell 58, 155–170. 36693321 10.1016/j.devcel.2022.12.007 PMC9904021

[56] Houbaviy HB , Dennis L , Jaenisch R and Sharp PA (2005) Characterization of a highly variable eutherian microRNA gene. RNA 11, 1245–1257. 15987809 10.1261/rna.2890305 PMC1370808

[57] Chakravarthy S , Sternberg SH , Kellenberger CA and Doudna JA (2010) Substrate‐specific kinetics of dicer‐catalyzed RNA processing. J Mol Biol 404, 392–402. 20932845 10.1016/j.jmb.2010.09.030 PMC3005596

[58] Ma E , MacRae IJ , Kirsch JF and Doudna JA (2008) Autoinhibition of human dicer by its internal helicase domain. J Mol Biol 380, 237–243. 18508075 10.1016/j.jmb.2008.05.005 PMC2927216

[59] Felix MA , Ashe A , Piffaretti J , Wu G , Nuez I , Belicard T , Jiang Y , Zhao G , Franz CJ , Goldstein LD *et al*. (2011) Natural and experimental infection of Caenorhabditis nematodes by novel viruses related to nodaviruses. PLoS Biol 9, e1000586. 21283608 10.1371/journal.pbio.1000586 PMC3026760

[60] Wang XH , Aliyari R , Li WX , Li HW , Kim K , Carthew R , Atkinson P and Ding SW (2006) RNA interference directs innate immunity against viruses in adult drosophila. Science 312, 452–454. 16556799 10.1126/science.1125694 PMC1509097

[61] Gantier MP and Williams BR (2007) The response of mammalian cells to double‐stranded RNA. Cytokine Growth Factor Rev 18, 363–371. 17698400 10.1016/j.cytogfr.2007.06.016 PMC2084215

[62] Demeter T , Vaskovicova M , Malik R , Horvat F , Pasulka J , Svobodova E , Flemr M and Svoboda P (2019) Main constraints for RNAi induced by expressed long dsRNA in mouse cells. Life Sci Alliance 2, e201800289. 30808654 10.26508/lsa.201800289 PMC6391682

[63] Seo GJ , Kincaid RP , Phanaksri T , Burke JM , Pare JM , Cox JE , Hsiang TY , Krug RM and Sullivan CS (2013) Reciprocal inhibition between intracellular antiviral signaling and the RNAi machinery in mammalian cells. Cell Host Microbe 14, 435–445. 24075860 10.1016/j.chom.2013.09.002 PMC3837626

[64] Takahashi T , Nakano Y , Onomoto K , Yoneyama M and Ui‐Tei K (2018) Virus sensor RIG‐I represses RNA interference by interacting with TRBP through LGP2 in mammalian cells. Genes (Basel) 9, 511. 30347765 10.3390/genes9100511 PMC6210652

[65] van der Veen AG , Maillard PV , Schmidt JM , Lee SA , Deddouche‐Grass S , Borg A , Kjaer S , Snijders AP and Reis e Sousa C (2018) The RIG‐I‐like receptor LGP2 inhibits dicer‐dependent processing of long double‐stranded RNA and blocks RNA interference in mammalian cells. EMBO J 37, EMBJ201797479. 10.15252/embj.201797479 PMC5813259 29351913

[66] Stein P , Zeng F , Pan H and Schultz RM (2005) Absence of non‐specific effects of RNA interference triggered by long double‐stranded RNA in mouse oocytes. Dev Biol 286, 464–471. 16154556 10.1016/j.ydbio.2005.08.015

[67] Karlic R , Ganesh S , Franke V , Svobodova E , Urbanova J , Suzuki Y , Aoki F , Vlahovicek K and Svoboda P (2017) Long non‐coding RNA exchange during the oocyte‐to‐embryo transition in mice. DNA Res 24, 129–141. 28087610 10.1093/dnares/dsw058 PMC5397607

[68] Kaneda M , Tang F , O'Carroll D , Lao K and Surani MA (2009) Essential role for Argonaute2 protein in mouse oogenesis. Epigenetics Chromatin 2, 9. 19664249 10.1186/1756-8935-2-9 PMC2736168

[69] Stein P , Rozhkov NV , Li F , Cardenas FL , Davydenko O , Vandivier LE , Gregory BD , Hannon GJ and Schultz RM (2015) Essential role for endogenous siRNAs during meiosis in mouse oocytes. PLoS Genet 11, e1005013. 25695507 10.1371/journal.pgen.1005013 PMC4335007

[70] Flemr M , Malik R , Franke V , Nejepinska J , Sedlacek R , Vlahovicek K and Svoboda P (2013) A retrotransposon‐driven dicer isoform directs endogenous small interfering RNA production in mouse oocytes. Cell 155, 807–816. 24209619 10.1016/j.cell.2013.10.001

[71] Franke V , Ganesh S , Karlic R , Malik R , Pasulka J , Horvat F , Kuzman M , Fulka H , Cernohorska M , Urbanova J *et al*. (2017) Long terminal repeats power evolution of genes and gene expression programs in mammalian oocytes and zygotes. Genome Res 27, 1384–1394. 28522611 10.1101/gr.216150.116 PMC5538554

[72] Xue Z , Huang K , Cai C , Cai L , Jiang CY , Feng Y , Liu Z , Zeng Q , Cheng L , Sun YE *et al*. (2013) Genetic programs in human and mouse early embryos revealed by single‐cell RNA sequencing. Nature 500, 593–597. 23892778 10.1038/nature12364 PMC4950944

[73] Ganesh S , Horvat F , Drutovic D , Efenberkova M , Pinkas D , Jindrova A , Pasulka J , Iyyappan R , Malik R , Susor A *et al*. (2020) The most abundant maternal lncRNA Sirena1 acts post‐transcriptionally and impacts mitochondrial distribution. Nucleic Acids Res 48, 3211–3227. 31956907 10.1093/nar/gkz1239 PMC7102984

[74] Petrzilek J , Pasulka J , Malik R , Horvat F , Kataruka S , Fulka H and Svoboda P (2022) De novo emergence, existence, and demise of a protein‐coding gene in murids. BMC Biol 20, 272. 36482406 10.1186/s12915-022-01470-5 PMC9733328

[75] Paradis F , Vigneault C , Robert C and Sirard MA (2005) RNA interference as a tool to study gene function in bovine oocytes. Mol Reprod Dev 70, 111–121. 15570624 10.1002/mrd.20193

[76] Anger M , Klima J , Kubelka M , Prochazka R , Motlik J and Schultz RM (2004) Timing of Plk1 and MPF activation during porcine oocyte maturation. Mol Reprod Dev 69, 11–16. 15278898 10.1002/mrd.20151

[77] Yan Z , Ma YZ , Liu DJ , Cang M , Wang R and Bao S (2010) Targeted suppression of Connexin 43 in ovine preimplantation embryos by RNA interference using long double‐stranded RNA. Asian‐Aust J Anim Sci 23, 456–464.

[78] Zapletal D , Taborska E , Pasulka J , Malik R , Kubicek K , Zanova M , Much C , Sebesta M , Buccheri V , Horvat F *et al*. (2022) Structural and functional basis of mammalian microRNA biogenesis by dicer. Mol Cell 82, 4064–4079. 36332606 10.1016/j.molcel.2022.10.010 PMC9645528

[79] Taborska E , Pasulka J , Malik R , Horvat F , Jenickova I , Jelic Matosevic Z and Svoboda P (2019) Restricted and non‐essential redundancy of RNAi and piRNA pathways in mouse oocytes. PLoS Genet 15, e1008261. 31860668 10.1371/journal.pgen.1008261 PMC6944382

[80] Wang X , Ramat A , Simonelig M and Liu MF (2023) Emerging roles and functional mechanisms of PIWI‐interacting RNAs. Nat Rev Mol Cell Biol 24, 123–141. 36104626 10.1038/s41580-022-00528-0

[81] Haase AD , Ketting RF , Lai EC , van Rij RP , Siomi M , Svoboda P , van Wolfswinkel JC and Wu PH (2024) PIWI‐interacting RNAs: who, what, when, where, why, and how. EMBO J 43, 5335–5339. 39327528 10.1038/s44318-024-00253-8 PMC11574264

[82] Cecchini K , Zamani M , Ajaykumar N , Vega‐Badillo J , Bagci A , Bailey S , Zamore PD and Gainetdinov I (2026) Cleavage of mRNAs by a minority of pachytene piRNAs improves sperm fitness. Nature 652, 508–516. 41639461 10.1038/s41586-026-10102-9 PMC13061629

[83] Loubalova Z , Ahrend F , Stoyko D , Cosby R , Ralls S , Meister G , Macfarlan T and Haase AD (2025) “Pachytene piRNAs define a conserved program of meiotic gene regulation: Pachytene piRNAs directly regulate select mRNAs by and RNAi‐like mechanism, establishing a regulatory paradigm of mammalian meiosis, *bioRxiv.”* .

[84] Carmell MA , Girard A , van de Kant HJG , Bourc'his D , Bestor TH , de Rooij DG and Hannon GJ (2007) MIWI2 is essential for spermatogenesis and repression of transposons in the mouse male germline. Dev Cell 12, 503–514. 17395546 10.1016/j.devcel.2007.03.001

[85] Deng W and Lin HF (2002) Miwi, a murine homolog of piwi, encodes a cytoplasmic protein essential for spermatogenesis. Dev Cell 2, 819–830. 12062093 10.1016/s1534-5807(02)00165-x

[86] Kuramochi‐Miyagawa S , Watanabe T , Gotoh K , Totoki Y , Toyoda A , Ikawa M , Asada N , Kojima K , Yamaguchi Y , Ijiri TW *et al*. (2008) DNA methylation of retrotransposon genes is regulated by Piwi family members MILI and MIWI2 in murine fetal testes. Genes Dev 22, 908–917. 18381894 10.1101/gad.1640708 PMC2279202

[87] Zheng K , Xiol J , Reuter M , Eckardt S , Leu NA , McLaughlin KJ , Stark A , Sachidanandam R , Pillai RS and Wang PJ (2010) Mouse MOV10L1 associates with Piwi proteins and is an essential component of the Piwi‐interacting RNA (piRNA) pathway. Proc Natl Acad Sci USA 107, 11841–11846. 20534472 10.1073/pnas.1003953107 PMC2900664

[88] Lim AK , Lorthongpanich C , Chew TG , Tan CW , Shue YT , Balu S , Gounko N , Kuramochi‐Miyagawa S , Matzuk MM , Chuma S *et al*. (2013) The nuage mediates retrotransposon silencing in mouse primordial ovarian follicles. Development 140, 3819–3825. 23924633 10.1242/dev.099184 PMC4067262

[89] Kabayama Y , Toh H , Katanaya A , Sakurai T , Chuma S , Kuramochi‐Miyagawa S , Saga Y , Nakano T and Sasaki H (2017) Roles of MIWI, MILI and PLD6 in small RNA regulation in mouse growing oocytes. Nucleic Acids Res 45, 5387–5398. 28115634 10.1093/nar/gkx027 PMC5435931

[90] De Fazio S , Bartonicek N , Di Giacomo M , Abreu‐Goodger C , Sankar A , Funaya C , Antony C , Moreira PN , Enright AJ and O'Carroll D (2011) The endonuclease activity of Mili fuels piRNA amplification that silences LINE1 elements. Nature 480, 259–263. 22020280 10.1038/nature10547

[91] Di Giacomo M , Comazzetto S , Saini H , De Fazio S , Carrieri C , Morgan M , Vasiliauskaite L , Benes V , Enright AJ and O'Carroll D (2013) Multiple epigenetic mechanisms and the piRNA pathway enforce LINE1 silencing during adult spermatogenesis. Mol Cell 50, 601–608. 23706823 10.1016/j.molcel.2013.04.026

[92] Lv X , Xiao W , Lai Y , Zhang Z , Zhang H , Qiu C , Hou L , Chen Q , Wang D , Gao Y *et al*. (2023) The non‐redundant functions of PIWI family proteins in gametogenesis in golden hamsters. Nat Commun 14, 5267. 37644029 10.1038/s41467-023-40650-x PMC10465502

[93] Casper J , Speir ML , Raney BJ , Perez G , Nassar LR , Lee CM , Hinrichs AS , Gonzalez JN , Fischer C , Diekhans M *et al*. (2026) The UCSC genome browser database: 2026 update. Nucleic Acids Res 54, D1331–D1335. 41251146 10.1093/nar/gkaf1250 PMC12807699

[94] Smallwood SA , Tomizawa S , Krueger F , Ruf N , Carli N , Segonds‐Pichon A , Sato S , Hata K , Andrews SR and Kelsey G (2011) Dynamic CpG Island methylation landscape in oocytes and preimplantation embryos. Nat Genet 43, 811–814. 21706000 10.1038/ng.864 PMC3146050

[95] Kumar S , Suleski M , Craig JM , Kasprowicz AE , Sanderford M , Li M , Stecher G and Hedges SB (2022) TimeTree 5: an expanded resource for species divergence times. Mol Biol Evol 39, msac174. 35932227 10.1093/molbev/msac174 PMC9400175

